# The evolving roles of alarin in physiological and disease conditions, and its future potential clinical implications

**DOI:** 10.3389/fendo.2022.1028982

**Published:** 2022-09-29

**Authors:** Endeshaw Chekol Abebe, Misganaw Asmamaw Mengstie, Mohammed Abdu Seid, Tabarak Malik, Tadesse Asmamaw Dejenie

**Affiliations:** ^1^ Department of Medical Biochemistry, College of Health Sciences, Debre Tabor University, Debre Tabor, Ethiopia; ^2^ Department of Physiology, College of Health Sciences, Debre Tabor University, Debre Tabor, Ethiopia; ^3^ Department of Medical Biochemistry, College of Medicine and Health Sciences, University of Gondar, Gondar, Ethiopia

**Keywords:** alarin, the galanin family, neuropeptides, physiological role, clinical implications

## Abstract

Alarin is a member of the galanin family of neuropeptides that is widely expressed in the central nervous system and peripheral tissues in humans and rodents. It was initially isolated fifteen years ago in ganglionic cells of human neuroblastoma. Subsequently, it was demonstrated to be broadly distributed in the blood vessels, skin, eyes, peripheral and central nervous systems, thymus, gastrointestinal tract, and endocrine organs of different species. Alarin is a 25 amino acid neuropeptide derived from the alternative splicing of the GALP gene, missing exon 3. It is found to be involved in several physiological functions that include feeding behavior, energy homeostasis, glucose homeostasis, body temperature, and reproduction. It has also vasoactive, anti-inflammatory, anti-edema, and antimicrobial activities. However, the physiological effects of alarin have not been fully elucidated and the receptors that mediate these effects are not currently known. Unearthing the novel biological effects of alarin and its unidentified receptors will therefore be a task in future biomedical research.

In addition, alarin is involved in various disease conditions, such as metabolic syndrome, obesity, insulin resistance, type 2 diabetes, diabetic retinopathy, hypertension, cardiac fibrosis, polycystic ovarian syndrome, and depression. Thus, alarin may serve as a promising tool for future pharmacological treatment and diagnosis. But further research is awaited to confirm whether alarin has a protective or pathological role in these diseases. This article provides a comprehensive review on the evolving implications of alarin in a variety of physiological and disease conditions, and its future perspectives.

## Introduction

Alarin is a recently discovered, 25-amino acid long, bioactive peptide of the galanin family (1). The galanin family is a pleiotropic group of neuropeptides with a wide range of distribution in the peripheral and central nervous systems (CNS). It was forty years ago that the parent peptide of the galanin family, known as galanin, was first discovered (2). Subsequently, other members of the galanin family, such as galanin message-associated protein (GMAP), galanin-like peptide (GALP), alarin, and spexin, were identified to have evolved *via* a series of gene duplications from a common ancestral peptide (3). Alarin was initially discovered in gangliocytes of human neuroblastic tumors by Santic and coworkers in 2006 (1). It was named ‘alarin’ from the amino acid alanine from the N-terminal end and serine from the C-terminal. It has currently been found to be highly expressed in the nervous system and a variety of peripheral tissues in various species (1, 4, 5).

The widespread distribution of alarin highlights its diverse range of physiological functions, which are related to its localization in a particular area of the body. Its expression in the brain is related to its central regulatory role in feeding behavior, energy homeostasis, glucose homeostasis, body temperature, and reproductive hormone secretion (5–7). Besides, its localization around the blood vessels of the skin and eye provides vasoactive, anti-inflammatory, anti-edema, and antimicrobial effects (4, 8, 9). Alarin is also indicated to be involved in various disease conditions, such as obesity, metabolic syndrome (MetS), insulin resistance (IR), type 2 diabetes (T2DM), diabetic retinopathy, hypertension, cardiac fibrosis, polycystic ovarian syndrome (PCOS), and depression (9–12). This review article discusses the involvement of alarin in different physiological and disease conditions., and its future perspectives. The information was gathered from various journals *via* electronic searches using PubMed, Google Scholar, Scopus, science direct, HINARI, and Cochrane Library from inception to 2022.

## Tissue distribution of alarin

Alarin has been demonstrated to be broadly distributed in the brain and peripheral tissues (**Table 1**). After being first isolated in the gangliocytes of neuroblastoma, alarin has then been identified to be expressed in the periphery, notably near the blood vessels of murine and human skin tissue (1, 4). Subsequently, it was found to be distributed in the central and peripheral nervous system, thymus, eyes, gastrointestinal tract, and several endocrine organs (4–6, 13–17). According to numerous rodent studies, the alarin peptide is widely expressed throughout the entire brain (5, 6, 13, 14). High-intensity alarin-like immunoreactivity (LI) was detected in a number of nuclei in the CNS of rodents, including the mitral cell layer of the olfactory bulb, the accessory olfactory bulb, the medial preoptic area, the amygdala, and the bed nucleus of the stria terminalis (13). In addition, several studies showed a markedly increased alarin-stimulated expression of the *c-fos gene* in different nuclei in the hypothalamus, including the paraventricular nucleus (PVN), dorsomedial nucleus (DMN), and the arcuate nucleus (ARC), ventromedial nucleus (VMN), and lateral hypothalamus (5, 6, 13). Alarin activity is also identified to be localized in the locus coeruleus (LC), and locus subcoeruleus of the midbrain and hindbrains, as well as the trigeminal complex, the ventral cochlear nucleus, the facial nucleus, and the epithelial layer of the plexus choroides in the brain (5, 13, 14). Similar to galanin, alarin has been discovered to have a significantly wider CNS expression pattern than GALP, including DMN, PVN, and different lateral nuclei of the hypothalamus (13).

**Table 1:** Table summary of the tissue distribution (expressions) of alarin.

**Studies**	**Study animal**	**Findings**
Santic et al., 2006 (1)	Human	A pioneering study isolated alarin for the first time from the gangliocytes of differentiated neuroblastic tumor tissues.
Santic et al., 2007 (4)	Murine	Alarin was expressed in the murine brain, thymus, and skin.
Van Der Kolk et al., 2010 (5)	Rats	Alarin immunoreactive cell bodies were detected within the LC and locus subcoeruleus of the midbrain. Alarin stimulated Fos induction in hypothalamic nuclei, such as the PVN and the nucleus of the tractus solitarious.
Fraley et al., 2012 (6)	Mice	Alarin-stimulated c-fos immunoreactivity was observed in diencephalic nuclei, including the hypothalamic DMN and the bed nucleus of the stria terminalis
Eberhard et al., 2012 (13)	Mouse	Alarin-LI was observed in different areas of the murine brain. High intensity of alarin-LI was detected in the accessory olfactory bulb, the medial preoptic area, the amygdala, different nuclei of the hypothalamus such as the ARC and VMN, the trigeminal complex, the LC, the ventral chochlear nucleus, the facial nucleus, and the epithelial layer of the plexus choroideus.
Eberhard et al., 2013 (14)	Human	Alarin is present in a variety of CNS nuclei as demonstrated from medium to high-intensity alarin-LI in all choroid plexus tumors, in the majority of ependymomas, and the minority of astrocytomas, meningiomas, and tumors of the cranial nerves. But alarin-LI was not detectable oligodendrogliomas and oligoastrocytoma.
Schrödl et al., 2013 (15)	Human, mouse, and rat	Alarin-LI was detected in ocular epithelial cells of the conjunctiva, cornea, and ciliary body; the blood vessels of the iris, retina, choroid, and neurons of the retina and human choroid.
Jabari et al., 2019 (16)	Human	Alarin is distributed in various enteroendocrine and Paneth cells and it may be involved in different physiological and pathological processes.
Tyczewska, et al., 2019 (17)	Rat	Alarin mRNA expression was observed in the hypothalamus, pituitary gland, and adrenal gland of the HPA axis.

Alarin-LI, alarin-like immunoreactivity; ARC, arcuate nucleus; CNS, central nervous system; HPA axis, hypothalamic-pituitary-adrenal axis; LC, locus coeruleus; PVN, paraventricular nucleus; VMN, ventromedial nucleus.

Schrödl et al. also found that alarin is widely distributed in the eyes of various species, including humans, mice, and rats. Alarin-LI was detected in ocular epithelial cells of the conjunctiva, cornea, and ciliary body; the blood vessels of the iris, retina, and choroid, as well as neurons of the retina and choroid (15). Immunohistochemical studies also observed alarin activity in various types of human intestinal epithelial cells, mainly enteroendocrine cells and Paneth cells (16). Recently, alarin mRNA was found to be expressed in the hypothalamus, pituitary gland, and adrenal gland of the hypothalamic-pituitary-adrenal axis (HPA) axis (17).

## Gene, structure, and biosynthesis of alarin

**Figure 1:**
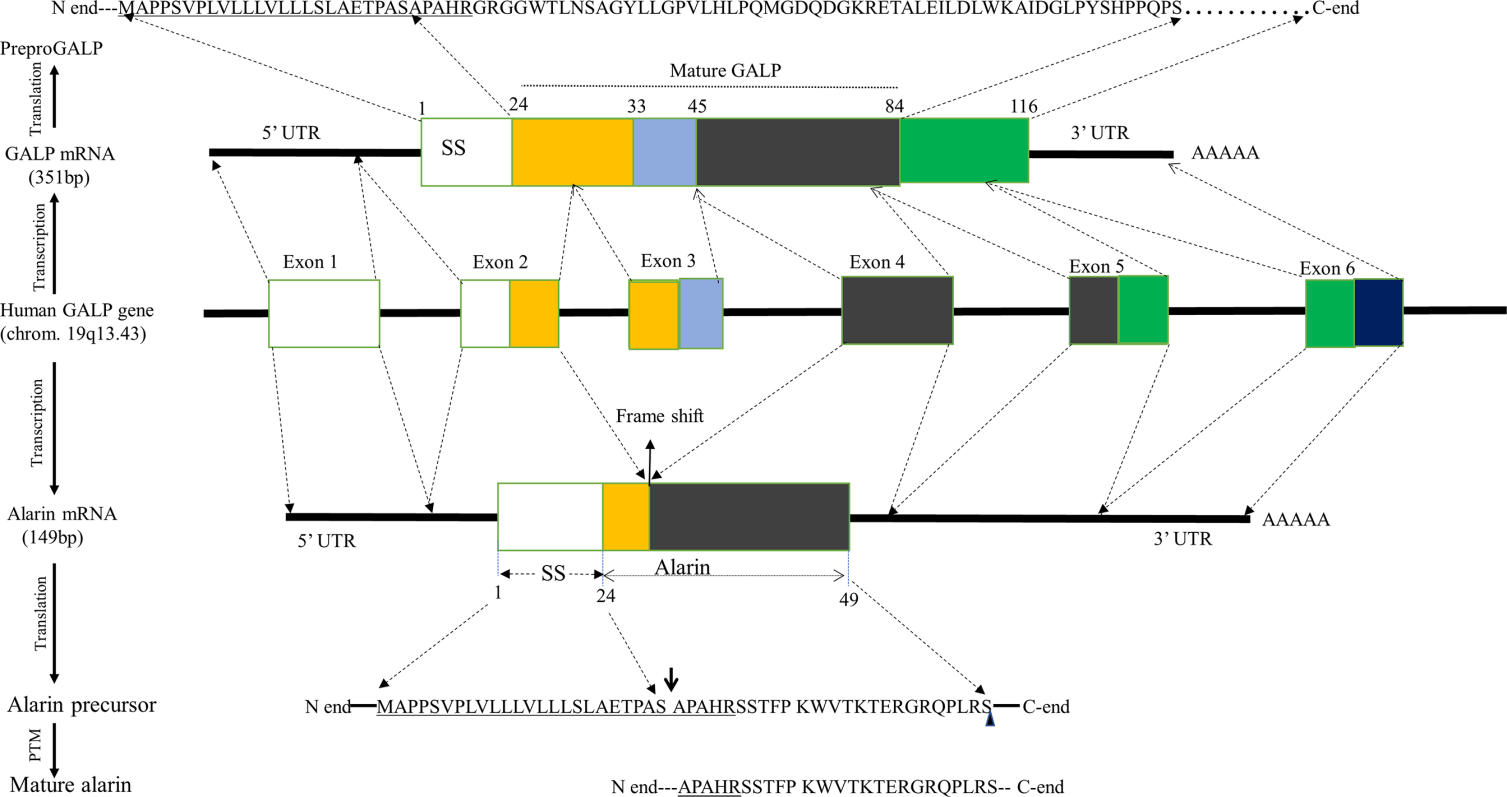
Schematic representation of human alarin biosynthesis. Human alarin is encoded by the GALP gene located on chromosome19q13.43. This gene undergoes transcription and alternative splicing by excluding exon 3, resulting in a frameshift to form alarin mRNA. The first, fifth, and sixth exons are noncoding, while exon 2 and exon 4 are coding sequences of alarin mRNA, which will be translated into a 49 amino acid precursor peptide. In alarin precursor, the underlined letters are the shared amino acid residues with the pre-proGALP, the downward arrow indicates its potential cleavage sites, and the small rectangular shape represents its site of amidation. Abbreviations: GALP, Galanin-like peptide; PTM, Posttranslational modification; SS, signal sequence; UTR, untranslated region.

Alarin is a short peptide that is evolutionary related to GALP, showing structural and functional similarities, since they are both encoded by the GALP gene (1, 6, 18). The GALP gene is a single-copy 11-kilobase sized gene organized into six small exons that are found on chromosome 19q13.43 in humans, chromosome 1q12 in rats, and chromosome 7A1 in mice (19, 20). The GALP gene exhibits extensive differential splicing in a variety of tissues (**Figure 1**) (4). Alternative post-transcriptional splicing generally plays a key role in increasing the diversity of gene products, known as proteomic diversity, including neuropeptides (21). Likewise, alarin mRNA is a splice variant of the preGALP mRNA derived by differential splicing of the GALP gene by skipping exon 3 that results in a frame shift, leading to a new peptide sequence and a stop codon after 49 amino acids (1, 3). Based on the interspecies comparison, a similar frame shift of the alarin splice variant has been observed in rats, macaques, and humans. However, there is no *in situ* hybridization or immunostaining so far that reports whether the GALP mRNA and alarin mRNA are colocalized in the same cells.

The GALP mRNA missing exon 3 (which is now alarin mRNA) is translated into an alarin precursor that comprises the same N-terminal end, including signal sequence (SS) and proteolytic cleavage site, as prepro GALP but a different C-terminal end (1, 4). The alarin precursor undergoes enzymatic processing after translation, but little is currently known about its post-translational modification. Of note, mature GALP is produced after the processing of pre-pro GALP by removing SS and endoproteolytic cleavage that is guided by basic amino acids flanking the mature peptides (20). Therefore, it is most likely that the same processing and secretion machinery can be employed for alarin as the N-terminal parts of the GALP and alarin precursor molecules are identical, bearing the same SS and proteolytic cleavage sites (4). Accordingly, it is postulated that the SS situated at the N-terminus of alarin precursor is proteolytically cleaved with signal peptidase and goes through several chemical modifications, such as amidation, to form a mature alarin peptide with 25 amino acids. Based on *in vivo* data, post-translational amidation most likely occurs at the C-terminal serine of the alarin peptide (4). Mature alarin shares the first five conserved amino acids (APAHR) at the N-terminus with mature GALP. But all the other 20 amino acid residues in the C-terminal region of alarin are not homologous to any other peptides, including GALP. Unlike GALP, alarin does not share structural homology with galanin (1, 22).

**Figure 2:**
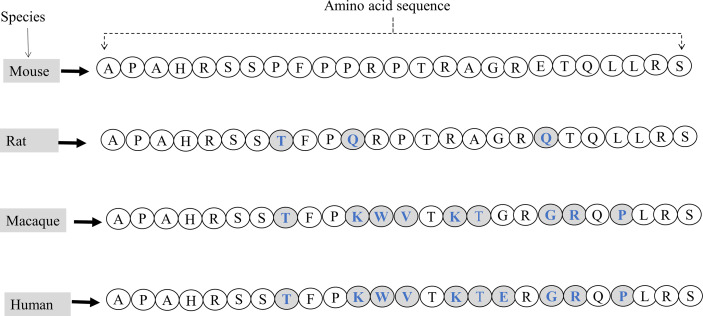
The amino acid sequences of the full-length mature alarin (containing 25 amino acids) and its comparisons in various species. Variations in amino acid sequences of alarin are observed in mice, rats, macaques, and humans. Letters written in blue in the shaded circles indicate the difference in amino acid sequence and composition of alarin compared to the mouse alarin.

Like GALP, alarin has been identified in various species, including rats, mice, macaques, and humans (4–6, 13–17). When compared to GALP, alarin shows a low degree of sequence similarity among species, which may indicate that alarin as an evolutionarily newer molecule (18). Among different species, alarin generally has a low degree of sequence similarity between primate and rodent (60%), while there is a high degree of sequence homology between murine and rat (92%), and humans and macaques (96%).The first seven amino acids of alarin are conserved between rodents and primates but the rest residues are different (**Figure 2**) (4).

## Physiological roles of alarin

**Figure 3:**
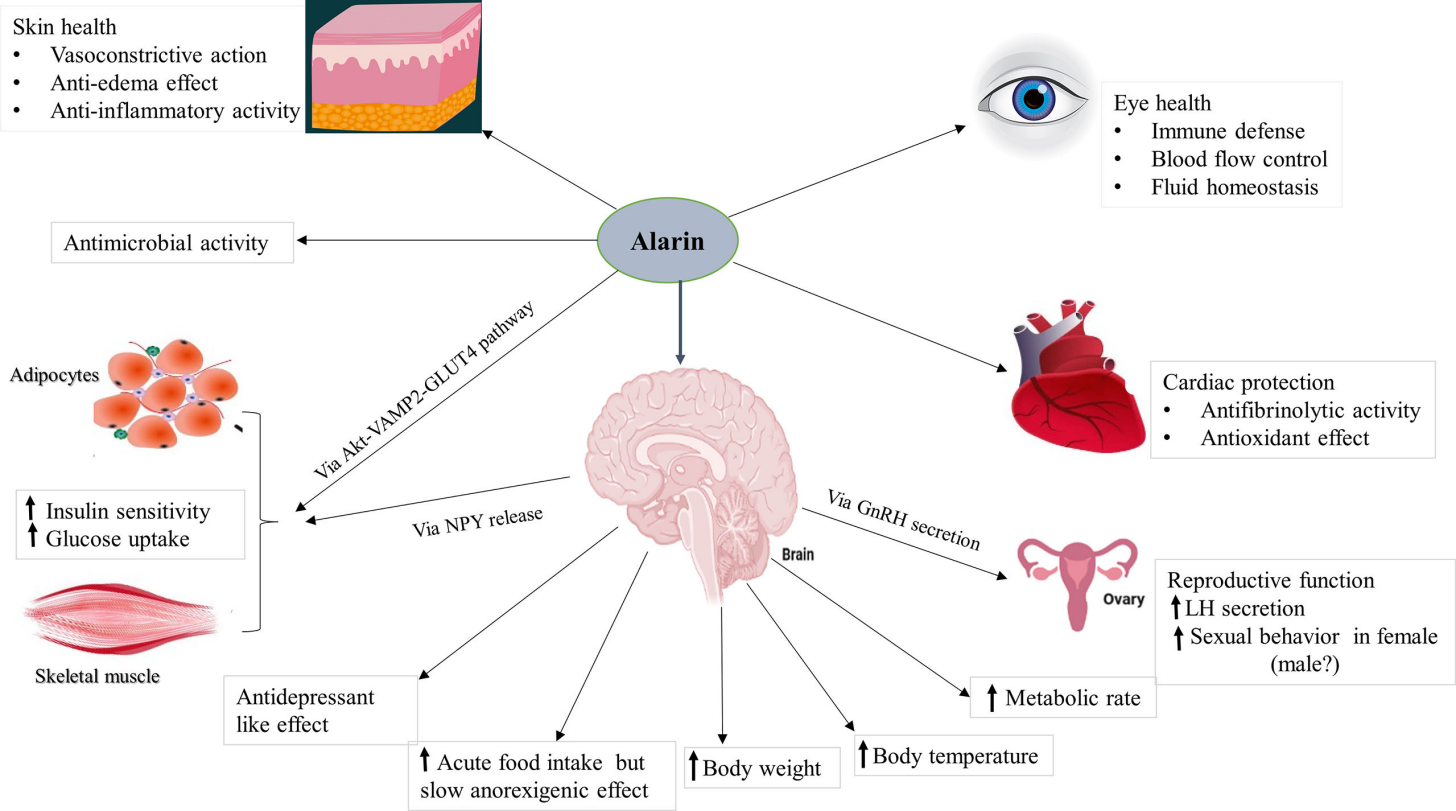
The putative functions of alarin. It is a pleiotropic peptide involved in various biological activities. Abbreviations: GLUT4, glucose transporter 4; GnRH, gonadotropin-releasing hormone; LH, luteinizing hormone; NPY, neuropeptide Y; VAMP2, vesicle-associated membrane protein 2.

Alarin is found to be a multifunctional peptide with a wide array of physiological functions that are generally related to its anatomical localization in a particular area of the body. It is found to regulate feeding behavior, energy homeostasis, glucose metabolism, body temperature, and reproduction. In addition, it has several other functions involving anti-inflammatory, vasoconstriction, and anti-edema effects to maintain eye and skin health. It also has antimicrobial activity against some bacteria. Unlike other members of the galanin family whose effects are mediated by three subtypes of galanin receptors (GAlR1, GalR2, and GalR3), the actions of alarin are not mediated by any of these receptors, and its receptors have not yet been defined (4, 7). This section of the review discusses the physiological functions of alarin based on the existing preclinical and clinical studies, which diagrammatically summarized in **Figure 3**.

### Regulatory effect on feeding behavior and energy homeostasis

Feeding behavior (food intake) is an essential component of energy homeostasis that provides all of the body’s nutrients (carbohydrates, lipids, proteins, minerals, and vitamins) (23). Energy homeostasis, which is also referred to as energy balance, is a biological process that entails the coordinated homeostatic regulation of energy inflow (food intake) and energy outflow (energy expenditure) (24). The central and peripheral mechanisms are critical in regulating energy homeostasis, including food intake. Importantly, food intake is centrally regulated by the interplay between many different hormones and neuropeptides, such as leptin, ghrelin, orexins, cerebelin, proopiomelanocortin (POMC), gonadotropin inhibitory hormone (GnIH), orexin, neuropeptide Y (NPY), neuropeptide W (NPW), galanin, and GALP (6, 25, 26). Alarin, as galanin and GALP, has recently been considered a regulatory peptide implicated in the control of feeding behavior and energy homeostasis. Several studies established that ICV injection (0.1-5.0nmol) of alarin into PVN significantly increases acute food intake (orexigenic) and body weight of animals (5–7, 18).

The orexigenic effect of centrally administered alarin in rats and mice has been observed to be acute (i.e., occurs before 24 hours) and short-lived (i.e., disappears after 3–4 hours). The food-stimulating effect of alarin was also demonstrated to have a dose-dependent response. Early studies indicated that while rats injected with 1.0 nmol and 5 nmol alarin had a significantly increased food intake after 30 minutes compared to the control, the doses of 0.1 nmol, 0.5 nmol, and 1.0 nmol only increased food consumption after between 2hours and 4 hours. But 5.0 nmol alarin did not further increase food intake, indicating the 1.0 nmol dose is the minimal dose that can elicit maximal effects before 24 hours (5, 7). This alarin-induced orexigenic effect suggested to be a result of its modulating effect on the autonomic activity of the gut. Furthermore, alarin has been postulated to exert orexigenic action by interacting with other neuromodulators, such as NPY, that are involved in the regulation of energy homeostasis. Alarin seems to stimulate food intake, partially through NPY release, as indicated by dramatically increased NPY secretion from the ARC after alarin treatment of hypothalamic explants (5, 7). Alarin injection (1.0 nmol) significantly increases the body weight of rodents after 24 hours in addition to food intake, most likely as a result of increased food intake and, to a lesser extent, increased water intake, decreased excretion of feces and urine, and increased metabolic rate (5–7).

Although more studies are required to explore the precise regions of the brain involved in the orexigenic activities of alarin, the strong expression of alarin-LI in a variety of brain nuclei is associated with the central nervous control of food intake, such as the ARC, DMN, PVN, and the lateral hypothalamic area, suggesting that these brain structures may be involved in the stimulatory effect of alarin on food intake (5–7, 13, 18). Some reports observed alarin immunoreactive cell bodies in the ARC of the hypothalamus in rats and mice and confirmed the possible involvement of this nucleus in mediating the effect of alarin in feeding behavior and energy homeostatic control (5, 13, 27). In addition, several studies reported that alarin expression appears to be localized to the hypothalamic DMN and within the LC and locus subcoeruleus of the midbrain, revealing that these areas may have a central role in coordinating metabolic feeding (5, 6).

In contrary, alarin has been shown to have slow anorexigenic character in other experimental settings. According to Miko et al. (2017), centrally injected alarin (3μg) at the onset of the active nighttime period slowly but strongly suppressed spontaneous nighttime cumulative food intake with no significant compensation in the following daytime period. Its administration after 24-hour fasting considerably decreased re-feeding food intake, and this anorexigenic effect persisted for up to 24 hours (28). This however requires further studies that clearly elucidate the timing, the required alarin dose to elicit anorexia, as well as the mechanism underlying it.

On the basis of the existing evidence, the regulatory role of alarin in feeding behavior demonstrates similarities and differences when compared to other neuropeptides, such as NPY and other members of the galanin family. Although alarin mediates its food stimulating effect *via* NPY, it appears to have a less potent orexigenic effect than NPY (18). The prompt orexigenic activity of alarin shows patterns somewhat different from those of NPY, but shares similarities in later phases. In contrast to NPY, which induces an acute increase in daytime spontaneous food intake followed by a rebound reduction in cumulative 24-hour food intake, alarin did not induce food intake during the daytime period, while also leading to a decrease in cumulative 24-hour food intake based on the animal study by Miko et al. in 2017 (28, 29). Moreover, the immediate food stimulating effect of alarin resembles the orexigenic effects of both galanin and GALP in most species, making acute orexigenic effects a common thread among all the members of the galanin peptide family (3, 30, 31). Similar to galanin and GALP, alarin has regulatory roles in orexigenic activity and energy metabolism, increasing food intake and body weight in animal models (5, 7, 18). The dose of alarin exerting an effect on feeding is in similar ranges to the effective dose of either GALP (1μg-10μg) or galanin (0.1 or 0.3 nmol) (32–35).The activity of alarin at higher concentrations (5 nmol dose) is lost, as seen in both galanin and GALP (5). This is known as the “ceiling” phenomenon, which is a well-known characteristic of neuropeptide pharmacology and exhibits a bell-shaped dose-response curve (36). Furthermore, alarin and GALP similarly produce a food intake stimulating effect by inducing NPY release from hypothalamic explants in the short term, while they slowly elicit anorexigenic effects (7). But alarin’s effects on food intake and body weight after 24 hours are different from those of GALP (32). GALP is found to exert anorexigenic effects over the longer term in rodents, whereas alarin does not affect feeding behavior at all or induces a slow anorexigenic effects after 24-hour. Additionally, GALP causes weight loss in both animals, in contrast to the effect of alarin, which results in a gain in body weight in rodents after 24 hours of injection (34, 35, 37, 38). \

### Thermoregulatory effects of alarin

Thermoregulation, which is another fundamental aspect of energy homeostasis, is defined as the ability to balance between heat production and heat loss to maintain a relatively constant core body temperature within a certain normal range (39). Many neuropeptides like galanin and GALP that regulate food intake also have effects on metabolic rate, body temperature, and energy expenditure (32, 38, 40). Numerous studies have also been conducted to identify the effects of the lately discovered alarin on body temperature and metabolic rate. Initially, a few studies indicated that alarin does not appear to have any effects on regulating either body temperature or metabolic rate, in contrast to galanin and GALP (5–7). A study by Van Der Kolk et al. (2010) indicated that ICV injection of alarin (1.0nmol) showed no significant change in oxygen consumption (or metabolic rate) in adult male freely moving Long-Evans rats (5). Fraley et al. (2012) also demonstrated that central alarin treatment (1.0nmol) did not show any significant change in body temperature in freely moving mice using biotelemetry (6).

But later on, a study by Miko and colleagues in 2014 revealed the significant thermoregulatory effect of alarin in rats (37). The involvement of alarin in food intake considers it an orexigenic and presumably anabolic galanin family member, implying that this peptide may have been anticipated to result in an inhibition of energy expenditure (thermogenesis) and temporal hypothermia (5–7). However, they demonstrated that centrally administered alarin (1, 3 or 15 μg) appears to be a catabolic mediator that elicits a slow but significant fever-like hypermetabolic/hyperthermic thermoregulatory response in rats without any initial hypothermia, even at a cooler (15°C) ambient temperature (37). Moreover, another independent study by Miko et al. in 2017 confirmed alarin as a central catabolic peptide that results in hyperthermic and hypermetabolic effects. It has been identified to induce a slow anorexigenic and prostaglandin-mediated, fever-like hyperthermic response in rats. The study added that alarin can also have a potential involvement in the disease’s behavior (28). In contrast, similarly to food intake-related observations, neither peripheral administration of full-length, biologically active alarin nor ICV injections of the truncated alarin peptide (Ala6-25Cys) has evoked any thermoregulatory responses (6, 18).

The dynamics of the thermoregulatory changes induced by alarin resembles its slow anorexigenic responses. The acute thermoregulatory effects of alarin show variations from those of NPY with similarities in later phases (28). In contrast to ICV injected NPY, which causes acute hypothermia with hypometabolism in a cool environment before leading to late hyperthermia, alarin induced a slow but marked fever-like hypermetabolic/hyperthermic response in a cool ambient temperature (28, 29, 37). Moreover, NPY does not induce vasoconstriction within a similar range of ambient temperatures (29). This warrants the need for further studies concerning the potential NPY mediation of alarin on thermoregulatory effects. On the other hand, the thermoregulatory response of alarin is similar to that of GALP. Upon acute injection of ICV GALP, the body temperature rises quickly, lasting 6-8 hours in rats. GALP also induces febrile effects over the longer term in rats and mice (34, 35, 38).The fever-like hyperthermia induced by GALP is mediated by interleukin (IL)-1 and prostaglandins, while the hypermetabolic effects result from the suppression of heat loss in rats (32, 40–42). Due to the similarities between the thermoregulatory actions of alarin and GALP as well as the delay in the onset of alarin-hyperthermia, prostaglandins are possibly involved in secondary mediation of the alarin-evoked effects. This suggests the potential function of alarin in inflammation, which could readily contribute to inflammation-induced fever (7, 28).

In general, alarin has a complex and opposing role in the regulation of energy homeostasis, including its acute and long-term effects on food intake, metabolic rate, and body temperature. Therefore, additional investigations are needed to understand the intricate role of alarin in energy balance and thermoregulation and thus reconcile the conflicting results and draw a conclusion. Moreover, more research is required to determine the various factors that regulate alarin release as well as those that mediate its effects.

### Regulatory role of alarin in glucose homeostasis

Alarin is an adipokine that plays a functional role in insulin-mediated glucose uptake in a variety of tissues in rodents (6, 7, 43). Numerous preclinical studies have been undertaken to evaluate the influence of alarin on the regulation of glucose metabolism. According to Guo et al. (2014), central injection of alarin significantly increases 2-(N-7-nitrobenz-2-oxa-1, 3-diazol-4-yl) amino-2-deoxyglucose uptake, plasma adiponectin levels, glucose infusion rates in hyperinsulinemic-euglycemic clamp (HEC) tests, vesicle-associated membrane protein 2 (VAMP2) as well as glucose transporter receptor 4 (GLUT 4) protein and mRNA expression, and the ratios of GLUT4 contents in plasma membranes to total cell membranes in adipocyte. But the same study reported an alarin-mediated decrement in plasma glucose and retinol-binding protein 4 (RBP4) levels in male rats (44). Zhang et al. (2015) also found that ICV release of alarin is significantly associated with increased glucose influx, glucose infusion rates in HEC tests, GLUT4 mRNA expression, GLUT4 translocations, Akt phosphorylation, GLUT4 and VAMP2 at the plasma membranes of the muscle cells, while blood glucose and insulin levels have been reduced (43). This suggests that central administration of alarin enhances glucose uptake of the skeletal muscles in T2DM rats (45).

Although the precise mechanism underlying the effect of alarin on glucose uptake and insulin sensitivity remains enigmatic, several putative mechanisms have been proposed. Alarin suggested promoting cellular glucose uptake and insulin sensitivity by increasing the GLUT4 content and its translocation from intracellular pools to plasma membranes of adipocytes and muscles (43, 44). Insulin-mediated glucose uptake, which is a crucial step in maintaining body glucose homeostasis, requires the assistance of the glucose transporter family, most notably GLUT4 (46). Alarin therapy of type 2 diabetic mice has been shown to increase GLUT4 and activate the Akt signaling pathway, thereby improving insulin sensitivity and glucose uptake (43, 44). Alarin-mediated activation of the Akt-VAMP2-GLUT4 pathway in the skeletal muscle is reported to enhance the translocation of GLUT4 from intracellular to cell membranes and thus increase glucose uptake, as evidenced by increased pAktThr308, pAktSer473, and total Akt levels after central injection of alarin (43). Similarly, the alarin–VAMP2–GLUT4 pathway is proposed to induce the beneficial effect of central alarin on glucose uptake and insulin sensitivity in adipocytes of diabetic animals (44).

Additionally, it has been discovered that the effects of alarin on insulin sensitivity can be mediated by decreasing RBP4 and raising adiponectin levels (44). Alarin is also postulated to regulate glucose uptake in muscles, at least in part, by regulating the activity of the hypothalamic-pituitary-gonadal axis. Moreover, alarin-stimulated increased c-Fos activity in the hypothalamus, diencephalon, as well as in the nucleus tractus solitarius and other centers of the hindbrain (rhombencephalon) suggests that alarin in the brain may regulate the release of many central transmitters, such as NPY, and influence glucose uptake in addition to food intake (6, 9). Alarin-mediated pleiotropic NPY release from hypothalamic explants directly enters into the adipose tissue by activating NPY-Y2 receptors to influence GLUT4 mRNA expression in adipocytes of subjects as well as to accelerate glucose intake that stimulates appetite and obesity (5, 47).

### Reproductive hormone secretion

Alarin is considered a neuromediator involved in the regulation of reproductive hormone secretion (5, 7). In animal experiments, alarin has been demonstrated to play an endocrine function by stimulating the release of sex hormones by regulating the activity of the hypothalamic-pituitary-gonadal axis in rats. Similarly to other members of the galanin peptide family such as galanin and GALP, several animal-based studies indicated that alarin promotes the secretion of luteinizing hormone (LH) and hypothalamic gonadotropin-releasing hormone (GnRH) (5–7, 18). Boughton et al. (2010) found that alarin has a potent property to stimulate the release of GnRH in both hypothalamic explants and a hypothalamic cell line in the male rat, which increases circulating LH levels (7). In another study, alarin injection (1.0nmol)resulted in an increase in serum LH concentration in castrated male rats, while there was no such effect in intact male rats (5). Besides, a study by Fraley et al. (2012) demonstrated that alarin-mediated LH secretion is significantly increased in a GnRH-dependent manner, but this alarin-specific effect is abolished by a truncated alarin inhibitor (Ala6-25Cys) of the putative alarin receptors (18). This is further confirmed by a prospective study, indicating a positive correlation between serum alarin and LH concentrations in infertile women with poor ovarian reserve (48).

Together, these findings suggest that alarin has important reproductive functions in females by inducing pre-ovulatory GnRH and LH surges and promoting sexual behavior in female rats (48). Although LH secretion is substantially increased by alarin injection in male rats, it does not affect male sexual behavior in intact or castrated rats (7). Consistently, Van Der Kolk et al. (2010)also proved that alarin has no effect on male sex behavior or Fos activation within the medial preoptic area (5). This alarin’s effect on male sexual effects is contradictory to the effects of galanin and GALP (34, 49). ICV injection of galanin (0-500ng) significantly reduces male-type sex behavior in rats, but when injected directly into the medial preoptic area, it stimulates male sexual behavior (49). On the other hand, central administration of GALP (15 nmol) is found to stimulate Fos expression in the medial preoptic area and markedly increase male sex behaviors (50, 51). However, Hu and collaborators have recently highlighted the distinct sexual dimorphism of circulating alarin, with a higher level in males, implying that this peptide may have gender-specific activity and/or be regulated by sex hormones (52). Therefore, more extensive research regarding the sexual dimorphism of blood alarin and its effect on sexual behavior is required to arrive at a conclusion.

### Role of alarin in skin health

Alarin has been proven to have vasoconstrictive, antiedema, and anti-inflammatory activities in the skin (4). Alarin is present in the skin in analogy to its ancestral members of the galanin family (galanin, GMAP, GALP, Spexin). Alarin expression in the periphery was initially described in the blood vessels of murine and human skin tissue, indicating its role in skin health. In the dermal vascular system, alarin-LI has been observed in the pericytes of microvascular arterioles and venules as well as in the layers of smooth muscle cells in larger vessels. Subsequent *in vivo* experimental results revealed that subcutaneous alarin injection in the skin was demonstrated to have potent and dose-related vasoconstrictive, anti-edema, and anti-inflammatory effects in the cutaneous microvasculature (4).

Under physiological conditions, alarin-mediated vasoconstriction is most likely due to neuronal input. Whereas the profound inhibitory effect of alarin on inflammatory edema formation could be due to a reduction of cutaneous blood flow due to its vasoconstrictor function as well as by lowering plasma permeability (1, 4). Furthermore, the vascular localization of alarin in the skin also exhibits anti-inflammatory effects to prevent substance P-induced neurogenic inflammation by vasoconstriction in the murine skin. The N-terminal end of alarin is thought to have an anti-inflammatory role, as evidenced by the reduction in its effectiveness in inhibiting neurogenic inflammation after the first two amino acids were removed (4, 18). It has been suggested that alarin may act *via* a new regulatory circuit to fine-tune the anti-inflammatory activity in order to maintain the homeostasis of the external barrier of the human body (4).

### Role of alarin in eye health

A recent study discovered alarin in various ocular tissues and indicated its involvement in ocular health (10, 15). Schrödl and coworkers have shown that there is a wide distribution of alarin in the eyes of various species, including humans, mice, and rats. Alarin-LI has been detected in ocular epithelial cells (conjunctiva, cornea, ciliary body), ocular blood vessels (iris, retina, choroid), and neurons (retina, choroid) (15). It is found in the iris of all species, in the corneal epithelium and endothelium of humans, mice, and rats, as well as in the conjunctiva of mice and rats. In human eyes, strong alarin-LI is also seen around retinal blood vessels and in intrinsic choroidal neurons (ICNs) (8, 15). This peptide has been demonstrated to play a critical role in eye health, including ocular immune defense, blood flow control, and fluid homeostasis. The existence of alarin in the cornea and conjunctiva indicates its role in immune defense, while its presence in the non-pigmented ciliary epithelium favors its involvement in aqueous humor production. The localization of alarin in the iris blood vessels and ciliary body further supports its role in the eye’s immune defense and the maintenance of the function of the corneal endothelium in the anterior chamber, with neurotransmitter and neuropeptide-like activities (15).

In addition, the activity of alarin detected around the retinal and choroidal blood vessels may be an indicator of its vasoactive or regulatory role in ocular blood flow. The potential effects of alarin on ocular blood vessels are supported by its dose-dependent vasoconstriction, anti-edema, and anti-inflammatory effects after subcutaneous injection into the skin (4). The vasoactive actions of alarin in blood vessels of the eyes are suggested to be a key player in regulating the vessel diameter and ocular blood flow, and therefore ocular fluid homeostasis (8). Vessel-specific ICNs are the proposed origins of alarin action in the choroidal blood vessels (15). The presence of alarin in the ICNs, which terminate in choroidal blood vessels, suggests its involvement in the regulation of choroidal blood flow that may have an impact on retinal hemostasis (15, 53). Besides, alarin in ICNs may act as a neuropeptide in combination with other neurotransmitters and neuropeptides, revealing that it may have neuromodulator activity in the choroid and retina (4, 15). Although extrinsic choroidal neurons play a role in modulating choroidal blood vessels, there is no evidence whether alarin mediates its vasoconstrictive role *via* these external sources of neurons supplying the choroid.

### Antimicrobial activity of alarin

Alarin has been identified as an antimicrobial peptide (AMP) that has an antimicrobial role against some bacteria (9). AMPs are peptides that inhibit microbial growth by interacting with the cell membranes and disrupting them. Several studies have recently been conducted to extensively explore AMPs to substitute the function of available antibiotics and treat resistant infectious agent (54). Human cathelicidin LL-37 (37aa), defensins (29–42aa), and histatins (32aa) are some of the extensively studied AMPs that hold the hope of replacing the role of antibiotics in the future (55). It has been discovered that members of the galanin neuropeptide family, such as galanin and GMAP, are potent AMPs against certain microbes. The available evidence shows that galanin is essential for the ability of the innate immune system to control infection caused by *Mycobacterium marinum (M. marinum*) and *Staphylococcus aureus (S. aureus)* in the larvae of zebrafish.56 While GMAP has growth inhibition activity against *C. albicans* and inhibits the transition from budded to hyphal form.57 Another study also corroborated that GMAP has a high potency against clinically relevant nonalbicans Candida strains such as *C. krusei, C. tropicalis and C. utilis*, establishing GMAP as a possible new component of the innate immune system (56).

Unlike GALP, alarin is also recently reported to exhibit a specific antimicrobial activity by dose-dependent inhibition of the growth of gram-negative bacteria, such as strain ML-35 of *Escherichia coli* (*E. coli*) *(* 9). The antimicrobial potency of alarin against *E. coli* has been shown to be comparable with another AMP called human cathelicidin LL-37. However, in contrast to human cathelicidin LL-37, alarin is not active against gram-positive bacteria like *S. aureus*. Alarin exerts antimicrobial activity against *E. coli* through bacterial membrane blebbing or disruption with no erythrocyte hemolysis, meeting the desirable characteristics of an effective AMP (9). According to Wada et al., the conserved amino acid sequence (APAHR) in the N-terminal region is thought to be crucial for the antimicrobial activity of alarin. Its antibacterial activity was reduced when the conserved APAHR sequence in the N-terminal region was deleted. This is also supported by the antimicrobial action of alarin, in contrast to GALP. In addition, the original sequence of its C-terminus (Ala6–25Cys) that contains certain basic amino acids is crucial for the antimicrobial actions of alarin, as demonstrated by the gradual reduction and eventual elimination of alarin’s antibacterial activity in subsequent deletions of C-terminal sequences. This shows that, while the APAHR sequence of alarin is required at the N-terminal end for its maximum antimicrobial activity, the C-terminus of alarin still provides the antimicrobial activity (9). Collectively, these findings demonstrate the therapeutic potential of alarin to be used as an antimicrobial and considered in the development of human therapeutics in the future.

## Role of alarin in various disease conditions

A growing body of evidence reveals that alarin appears to play roles, which can be protective or pathological, in some disease conditions, including obesity, MetS, IR, T2DM, cardiac fibrosis, hypertension, PCOS, and depression. This part of the review highlights the role of alarin in various disease conditions.

### Obesity and metabolic syndrome

Currently, accumulated evidence indicates that alarin may be implicated in the development of obesity and MetS (52, 57, 58). There is also evidence that alarin may have an involvement in the development of obesity. The effect of alarin on obesity is demonstrated by a significantly higher level of circulating alarin in obese children than in the healthy control group (58). Similarly, significantly elevated plasma alarin levels were seen in obese T2DM than in the non-obese T2DM group (57). Overweight subjects also showed elevated circulating levels of alarin when compared to normal weight subjects (59). Several studies also showed that the blood level of alarin is positively correlated with anthropometric measures of obesity, such as BMI, waist circumference, and hip circumference (45, 58, 60). Further, a significant correlation of alarin levels with adverse lipid profiles has been demonstrated. This suggests that alarin may be associated with the development of obesity, possibly through its orexigenic actions that increase appetite and body weight (45).

MetS, which is characterized by central obesity, high blood pressure, high triglyceride levels, low HDL cholesterol levels, and IR, has been shown to have an independent association with the circulating levels of alarin. It has also been reported that patients with MetS have significantly higher blood alarin levels than healthy subjects (45). This is further supported by another human study, which indicates that blood alarin levels are higher in women with MetS than controls (61). Alarin was shown to have a positive correlation with fasting blood glucose (FBG), body mass index (BMI), waist circumference, blood pressure, triglyceride, total cholesterol, glycosylated hemoglobin (HbA1c), homeostasis model assessment of β-cell function (HOMA-β), and HOMA of IR (HOMA-IR) in patients with MetS (45). Other studies have also found positive correlations between serum levels of alarin and BMI, waist circumference, blood pressure, triglyceride, FBG, HbA1c, fasting insulin, and HOMA-IR (58, 61). Furthermore, alarin was identified as an independent predictor of the risk factors of MetS, including central obesity, IR, dyslipidemia, hyperglycemia, and hypertension (45). Thus, the collective evidence indicates that alarin may be a contributory factor in the development of MetS.

### Insulin resistance and type 2 diabetes mellitus

Several data suggest that alarin may potentially play a pathological role in the emergence of IR. It has been indicated that alarin has a negative correlation with the whole-body insulin sensitivity index (WBISI), while it has a positive correlation with HOMA-IR (45). Besides its circulating levels are higher in the IR group than in the non-IR group (58). In agreement with this, alarin levels are elevated in subjects with IR when compared to those without IR (59). Moreover, oral infusion of glucose into healthy male individuals results in increased circulating alarin levels, whereas acute hyperinsulinemia transiently decreases serum alarin levels, suggesting the potential role of alarin in the progression of IR (45). Other studies also indicated a significant positive correlation between the circulating levels of alarin with plasma tumor necrosis factor-alpha (TNF-α) (45, 52). Based on the pre-existing notion that chronic inflammation underpins the development of IR, it is speculated that alarin is a cytokine that enhances the occurrence of IR (62, 63). However, further research into the relationship between alarin and IR is required to establish the role of alarin in IR.

Moreover, a plethora of studies found markedly increased plasma levels of alarin in T2DM subjects compared to controls. According to Zhou et al. (2021), alarin levels were observed to be elevated in newly diagnosed T2DM patients compared to control groups (57). Hu et al (2019) consistently found that the concentration of circulating alarin is significantly higher in both patients with impaired glucose tolerance (IGT) and T2DM than in healthy individuals. But plasma levels of alarin in T2DM patients were considerably greater than in subjects with IGT, demonstrating a progressive increment of circulating alarin from a pre-diabetic state to a diabetic stage (52). These findings may highlight the linkage of alarin to the occurrence and development of T2DM.

Notwithstanding the evidence, many scholars speculate that the elevation of circulating alarin in patients with T2DM could be a compensatory up-regulation for counteracting or adapting to the metabolic stress produced by abdominal obesity, IR, dyslipidemia, hyperglycemia, and hypertension (8). In addition, high alarin levels in T2DM subjects may be related to resistance to alarin action in a similar mechanism to insulin or leptin resistance, which results in increased alarin secretion (45, 52, 57). These hypotheses are supported by several *in vivo* studies that documented the beneficial effects of alarin on ameliorating IR, insulin levels, and blood glucose in T2DM (43, 44). Significantly elevated serum alarin levels have been observed in healthy control groups but not in T2DM groups (44). Other reports also demonstrated that central administration of alarin improved IR and glucose uptake in adipocytes of diabetic rats (43, 64). Taken together, these results indicate that alarin instead plays a beneficial antidiabetic role. Nevertheless, further large-scale studies are needed to determine whether alarin has a direct role in the development of IR/T2DM or serves as an early defensive response to metabolic stress. Besides, more research on the effect of alarin on the IR in apparently healthy and T2DM individuals is needed to reconcile the controversies.

### Diabetic retinopathy

Recently, alarin has also been reported to have implications for ocular diseases such as diabetic retinopathy. Gul et al. (2022) indicated that the concentration of both plasma and aqueous levels of alarin is significantly higher in patients with diabetic retinopathy than in the control group (8). This shows that alarin plays an important role in diabetic retinopathy, though it remains unclear whether alarin has a protective or contributing effect on the development of diabetic retinopathy. However, elevated levels of alarin in the aqueous humor are thought to be the result of a compensatory response to diabetic retinopathy or potential alarin resistance. High levels of alarin in diabetic retinopathy, which is associated with altered in retinal microvascular structure, macular edema, and inflammation, can also suggest that alarin is present at a level that is not sufficient to prevent the development of diabetic retinopathy (8). But extensive research is needed to conclusively address the role of alarin in diabetic retinopathy, as well as to determine whether the vasoconstrictor, anti-edema, and anti-inflammatory actions of alarin have a therapeutic value in diabetic retinopathy.

### Cardiac fibrosis

According to a recent study, alarin has a cardioprotective property that helps to prevent cardiac fibrosis in heart failure (HF). It was shown to improve cardiac dysfunction and attenuate cardiac fibrosis developed in HF rats, revealing that alarin serves as a potent cardioprotective agent similar to galanin (65). Some mechanisms have been proposed to explain how alarin plays an important role in interrupting the development of heart fibrosis in a rat model. Firstly, alarin could reduce cardiac fibrosis in HF rats by reducing the elevated levels of collagen and transforming growth factor-β (TGF-β) induced by angiotensin II (Ang II), thereby exerting antifibrotic activity. Secondly, alarin injection into cardiac fibroblasts may prevent fibrosis by alleviating oxidative stress in myocardial infarction (MI)-induced HF in rats, acting as an antioxidant (66). Alarin also attenuates cardiac fibrosis, possibly by reversing the elevated NADPH oxidase 1 (Nox1) activity, superoxide anion, and malondialdehyde (MDA) levels, as well as the reduced levels of superoxide dismutase (SOD) in the hearts of MI rats and Ang II-treated cardiac fibroblasts (65, 67). Given its cardioprotective role, alarin may be used as a promising tool for the treatment of HF in the future. However, further extensive studies elucidating the pharmacological and physiological roles of alarin in cardioprotective activity are needed.

### Hypertension

In addition, alarin has recently been discovered to be implicated in the pathogenesis of hypertension (11, 61). Numerous studies have reported the positive relationship between circulating alarin levels and blood pressure (45, 52, 57, 58, 61). Alarin has been found to increase levels of superoxide anions and NADPH oxidase activity in the hypothalamic PVN that modulates sympathetic activity and blood pressure and results in abnormal blood pressure or hypertension (68–73). Alarin also increases renal sympathetic nerve activity (RSNA), systolic blood pressure (SBP), diastolic blood pressure (DBP), and mean arterial pressure (MAP), which further contribute to hypertension development as well as progression to organ damage (11). Despite these results, additional research is necessary to confirm the involvement of alarin in the pathogenesis of hypertension.

### Polycystic ovarian syndrome

PCOS is accompanied by metabolic and endocrine abnormalities that disrupt pituitary-ovarian axis functions. Like metabolic syndrome, it is characterized by dyslipidemia, central obesity, and insulin resistance. Collective evidence demonstrates that the levels of circulating alarin in patients with PCOS were significantly elevated compared to controls (59, 61). The Turkish study was the first to confirm the association between serum alarin levels and different ovarian reserve patterns, including PCOS. There was a higher serum alarin concentration in the infertile women with PCOS and a positive correlation between serum alarin and LH level only in the PCOS women rather than other unexplained infertility (48, 61). Elevated levels of alarin is positively correlated with increased serum LH levels in women with PCOS. The same study also demonstrated that elevated serum alarin levels are associated with higher odds of having PCOS, suggesting alarin is an independent predictor of PCOS (61). Moreover, alarin is positively correlated with several parameters that are related to PCOS, such as IR marker, BMI, LH, and androgens. Its levels were significantly elevated in PCOS women with IR when compared to those without IR (58). The plasma levels of alarin are also substantially increased in overweight subjects compared to normal weight PCOS subjects. Because of the significant effect of alarin, like GALP, on neural elements that secrete GnRH and on deregulated gonadotropin production, it may potentially be implicated in the development of PCOS (59). Besides, alarin could be used as a new predictive marker of PCOS in the future.

### Depression

A number of recent studies have found that alarin, which is expressed in brain areas associated with depression such as the medial amygdala and hypothalamus, has an effect on depression-like behaviors (12, 27). Results from Wang and his coworkers revealed for the first time that alarin exhibits potent antidepressant-like effects both in the acute stress model and the unpredictable chronic mild stress (UCMS) depression-like model (27). Consistently, Zhuang et al. indicated that ICV administration of alarin significantly ameliorates depression-like behaviors in the UCMS mouse model (12). Subsequently, several other studies have confirmed the antidepressant properties of alarin (10, 74). Although the underlying mechanisms by which alarin mediates the antidepressant effect are still not clearly elucidated, several mechanisms have recently been suggested to mediate the antidepressant effect of alarin. Antidepressant-like effects of alarin may be mediated by targeting tropomyosin-related kinase B (TrkB), *via* the Ras-ERK and PI3K/AKT pathways, most likely by altering AKT, ERK, and CREB activity (10). The TrkB-activated Ras-ERK pathway, which is involved in cell proliferation, differentiation, apoptosis, and synaptic plasticity, and the TrKB-induced PI3K/AKT pathway, which regulates cell growth, survival, proliferation, and movement, improves neurogenesis, and exerts antidepressant-like effects (75–77).

Besides, alarin may exert its antidepressant effects by targeting mammalian target of rapamycin (mTOR), which stimulates ERK/AKT signaling pathways (12). Alarin could improve depression-like behaviors through the alarin-mediated restoration of the reduction of the phosphorylation of mTOR and its downstream substrates, eukaryotic initiation factor 4E binding protein 1 (4E-BP1) in the prefrontal cortex, hippocampus, hypothalamus, and olfactory bulb in depressive disorder. In such brain areas, alarin may also reverse depression-associated downregulation of ribosomal protein S6 kinase (p70S6K), post-synaptic density 95 (PSD-95), and pre-synaptic synapsin I protein expression (12). The restored synaptic proteins are essential for the formation, maturation, and function of synapses, thereby alleviating depression (78–83). Alarin is also postulated to have an antidepressant-like effect *via* the inhibition of the overactivated HPA axis and normalizing the serum levels of crucial glucocorticoid hormones (27, 84). Moreover, alarin may help alleviate depression by increasing the expression of brain-derived neurotrophic factor (BDNF) in the prefrontal cortex and hippocampus, which has antidepressant-like actions through neuroprotection and promoting synaptic plasticity (27).

## Future perspectives

Alarin peptide is widely dispersed throughout various tissues, from the brain to the peripheral tissues. However, the tissue distribution of alarin still calls for more thorough research to find additional alarin-expressing tissues and determine yet another function if any. In a manner similar to that of other proteins, the synthesis of alarin begins with GALP gene transcription and is followed by posttranscriptional differential splicing by excluding exon 3 of this gene to produce alarin mRNA and precursor peptide. Then, this precursor peptide will most likely undergo proteolytic cleavage of the SS and chemical modification to produce a 25-amino acid-containing active alarin. However, the posttranslational processing of alarin is still vague that warrants further research.

Although the entire roles of alarin remain unrevealed, it is a pleiotropic neuropeptide involved in a myriad of putative biological activities, adding a new dimension to the unusually high-functional redundancy of the galanin family. It has central regulatory effects on energy homeostasis, feeding behavior, glucose homeostasis, body temperature, and reproduction. Alarin also has vasoconstrictive, anti-inflammatory, and anti-edema activities in the skin and eye, conferring wellbeing for these tissues. It is also known to exhibit antibacterial activities, providing a new addition to the existing AMPs. Moreover, this peptide has been demonstrated to have a role in various disease conditions. It has been shown to have anti-fibrinolytic effects against cardiac fibrosis in HF rats, and antidepressant activity in UCMS rats. These pharmacological and physiological activities of alarin show that it could have potential use for the development of human therapeutics in the future.

Alarin has also been shown to have a putative role in the development of different diseases, like MetS, obesity, hypertension, IR dyslipidemia, and hyperglycemia. This new knowledge might be of utmost importance to use alarin as a possible diagnostic marker. On the other hand, the role of alarin in T2DM remains an area of debate, prompting more studies to reconcile the idea and reach a conclusion. Some reports documented that alarin is significantly higher in T2DM than controls, indicating alarin may have a pathogenic role in the progression of T2DM. But others suggest that increased alarin levels could be a compensatory up-regulation in response to metabolic abnormalities. On the contrary, several other studies showed that alarin increases insulin sensitivity and glucose uptake in T2DM rats, suggesting the antidiabetic activities of alarin. Increased knowledge of the antidiabetic role of the central alarin projective system reveals that alarin could be considered in the therapeutic arena in T2DM. The pharmacological activation of the alarin receptor and signaling, although not yet known, could modulate glucose metabolism and potentially serve as a therapeutic target for treating T2DM in the future.

But the other functions of alarin in the CNS and peripheral tissues remain obscure. Thus, many pieces of the puzzle regarding the biological functions of alarin remain to be uncovered. In spite of the fact that alarin is involved in various functions, the receptor that mediates its effects has not yet been identified. Research on the physiologic and pharmacologic characteristics of the alarin receptor is also still insufficient. However, recent evidence confirmed that alarin acts *via* a distinct receptor other than GalRs, leaving its receptors to be unveiled. This poses a new task of revealing its receptor on the basis of its pharmacological and biological activities as well as the mechanisms underlying these effects. A successful search for the alarin receptor will be necessary to draw valid conclusions on the putative biological functions of alarin.

## Concluding remarks

Alarin was first discovered over fifteen years ago as an alternate transcript of the GALP gene in neuroblastoma cells. It belongs to the galanin family and broadly distributes in the brain and peripheral tissues. Cumulative evidence indicated that alarin participates in numerous biological functions, including physiological and disease conditions. Consequently, alarin is nowadays attracting much interest from investigators and will have potential clinical implications for designing novel human therapeutic agents. The role of alarin in different diseases is also very promising for its future role as a diagnostic marker. However, its receptor is still unidentified though it is proven to act *via* a separate receptor other than GalRs. Therefore, thorough investigations are needed to investigate its receptors, validate these functions, and look for other moonlighting functions of alarin. In addition, further studies are also required to explore the pharmacological properties of alarin in detail.

## Author contributions

EA contributed to conceptualization and design of the review, article search, figure drawing, table preparation, and manuscript drafting. MS, MM, TM, and TD were involved in the preparation of the manuscript, tables, and critical revision. All authors contributed to the article and approved the submitted version.

## Conflict of interest

The authors declare that the research was conducted in the absence of any commercial or financial relationships that could be construed as a potential conflict of interest.

## Publisher’s note

All claims expressed in this article are solely those of the authors and do not necessarily represent those of their affiliated organizations, or those of the publisher, the editors and the reviewers. Any product that may be evaluated in this article, or claim that may be made by its manufacturer, is not guaranteed or endorsed by the publisher.

## Glossary

**Table:**

4E-BP1	eukaryotic initiation factor 4E binding protein 1
Alarin-LI	alarin-like immunoreactivity
AMPs	antimicrobial peptides
Ang II	angiotensin II
ARC	arcuate nucleus
BDNF	brain-derived neurotrophic factor
BMI	body mass index
CNS	central nervous system
CREB	cAMP Response Element-Binding Protein
DBP	diastolic blood pressure
ERK	extracellular signal-regulated kinase
FBG	fasting blood glucose
GALP	Galanin like peptide
GalRs	Galanin Receptors
GLUT4	glucose transporter 4
GMAP	galanin message-associated protein
GnIH	gonadotropin inhibitory hormone
GnRH	gonadotropin-releasing hormone
HbA1c	glycosylated hemoglobin
HDL	high-density lipoprotein
HEC	hyperinsulinemic-euglycemic clamp HF
heart failure	
HOMA-IR	Homeostasis Model Assessment of Insulin Resistance
HOMA-β	HOMA of β-cell function (HOMA-β)
HPA axis	hypothalamic-pituitary-adrenal axis
ICNs	intrinsic choroidal neurons
IGT	impaired glucose tolerance
IR	insulin resistance
LC	locus coeruleus
LH	luteinizing hormone
mTOR	mammalian target of rapamycin
MAP	mean arterial pressure
MDA	malondialdehyde
MetS	Metabolic syndrome
Nox1	NADPH oxidase 1
NPW	neuropeptide W
NPY	neuropeptide Y
p70S6K	ribosomal protein S6 kinase
PCOS	polycystic ovarian syndrome
PI3K	Phosphatidylinositol-3-kinase
POMC	Proopiomelanocortin
PVN	paraventricular nucleus
PSD-95	post-synaptic density 95
RBP4	retinol-binding protein 4
RSNA	renal sympathetic nerve activity
SBP	systolic blood pressure
SOD	superoxide dismutase
SS	signal sequence
TGF-β	transforming growth factor-β
TrkB	Tropomyosin-related kinase B
T2DM	Type 2 diabetes mellitus
UCMS	unpredictable chronic mild stress
VAMP2	vesicle associated membrane protein 2
VMN	ventromedial nucleus
WBISI	whole-body insulin sensitivity index
